# Clinical Yield of a Molecular Diagnostic Panel for Enteric Pathogens in Adult Outpatients With Diarrhea and Validation of Guidelines-Based Criteria for Testing

**DOI:** 10.1093/ofid/ofz162

**Published:** 2019-04-16

**Authors:** Stephen D Clark, Michael Sidlak, Amy J Mathers, Melinda Poulter, James A Platts-Mills

**Affiliations:** 1 Cecil G. Sheps Center for Health Services Research and Division of General Medicine and Clinical Epidemiology, Department of Medicine, University of North Carolina at Chapel Hill, Chapel Hill, North Carolina; 2 Clinical Microbiology, Department of Pathology, University of Virginia Health System, Charlottesville, Virginia; 3 Division of Infectious Diseases and International Health, University of Virginia, Charlottesville, Virginia

**Keywords:** diarrhea, guidelines, molecular diagnostics, PCR

## Abstract

**Background:**

Molecular diagnostic panels for enteric pathogens offer increased sensitivity and reduced turnaround time. However, many pathogen detections do not change clinical management, and the cost is substantial.

**Methods:**

We performed a retrospective chart review of adult outpatients with diarrhea at the University of Virginia who had samples tested by the FilmArray Gastrointestinal Panel (BioFire Diagnostics, Salt Lake City, UT) to identify the clinical yield and to validate the clinical criteria for testing recommended in the 2017 Infectious Diseases Society of America (IDSA) guidelines.

**Results:**

We analyzed 629 tests sent from adult outpatients with diarrhea between March 23, 2015, and July 18, 2016. A pathogen was detected in 127 of 629 specimens (20.2%). The most common pathogens were enteropathogenic *Escherichia coli* (47, 7.5%), norovirus (24, 3.8%), enteroaggregative *E. coli* (14, 2.2%), *Campylobacter* (9, 1.4%), and *Salmonella* (9; 1.4%). The clinical yield of testing was low, with antimicrobial treatment clearly indicated for only 18 subjects (2.9%) and any change in clinical management indicated for 33 subjects (5.2%). Following the clinical criteria for diagnostic testing from the 2017 IDSA guidelines, which suggest diagnostic testing for patients with fever, abdominal pain, blood in stool, or an immunocompromising condition, would have reduced testing by 32.3% without significantly reducing the clinical yield (sensitivity, 97.0%; 95% confidence interval [CI], 84.2%–99.9%; negative predictive value, 99.5%; 95% CI, 97.3%–100.0%).

**Conclusions:**

The clinical yield of molecular diagnostic testing in this population was low. Compliance with IDSA guidelines in adult outpatients with diarrhea could reduce testing by approximately one-third.

The evaluation and management of infectious diarrhea remain a common problem in the outpatient setting, with a broad range of etiologies accounting for 37.2 million cases per year in the United States [1]. Although most infectious diarrhea is self-limited, detection of the underlying pathogen can be of value where antimicrobial therapy would be indicated, where antimicrobial therapy should be explicitly withheld (ie, for shiga toxin–producing *Escherichia coli* [STEC]), when the patient’s degree of immunosuppression can be modified, and for public health reasons, for example, identification and control of outbreaks. The Infectious Disease Society of America (IDSA) published updated guidelines in October 2017 on the evaluation and management of infectious diarrhea [2]. With the exception of outbreak investigations, because most diarrhea is self-limited, these guidelines recommend restricting stool diagnostic testing to patients with either severe diarrhea (ie, signs of sepsis) or with fever, bloody or mucoid stools, severe abdominal cramping or tenderness, or an immunocompromising condition. This recommendation is supported by observational studies that have found an association between fever, abdominal pain, or blood and detection of a pathogen that would warrant a change in clinical management [3–5], although immunocompromised patients have the potential for severe and complicated disease or prolonged illness [2].

The detection of a broad array of potentially offending agents has traditionally required a combination of microbiologic approaches, including bacterial culture, antigen detection, microscopy, and polymerase chain reaction (PCR). However, these tests are time intensive, expensive, and in many cases the sensitivity is poor [6, 7]. Several Food and Drug Administration–approved molecular diagnostic panels have recently become available that offer increased sensitivity and reduced time to identification of pathogens while investigating for a broad range of causes [8, 9]. The IDSA guidelines recommend the use of these panels when testing is to be performed [2, 10]. However, the cost to the patient is significant, especially in the outpatient setting [10, 11]. Further, Medicare reimbursement for these tests has been restricted in the southeastern United States [12]. Our institution introduced the FilmArray gastrointestinal (GI) panel (BioFire Diagnostics, Salt Lake City, UT) in 2015, which tests for 20 enteropathogens. We sought to describe the clinical yield of testing in adult outpatients with diarrhea and to validate the IDSA recommendations for diagnostic testing in this population.

## METHODS

### Study Design and Population

We performed a retrospective chart review of adult patients who either presented to outpatient clinics or contacted outpatient clinic physicians by phone at the University of Virginia for whom a stool sample was tested by the FilmArray GI Panel (BioFire Diagnostics, Salt Lake City, UT) from March 23, 2015, to July 18, 2016. We excluded patients if there was no associated visit or telephone documentation within 72 hours of the test result if testing had been previously obtained for the patient within the review period, if the test was part of routine screening before fecal transplant, or if the patient did not have documented diarrhea.

For each subject, the following characteristics were abstracted from the clinical chart: diarrhea duration, visible blood in stool, subjective fever, and presence of associated enteric symptoms (abdominal pain, nausea, vomiting, bloating, fecal urgency, and tenesmus). If a characteristic other than duration was not described in the documentation, it was assumed that it was not present. Because telephone encounters were frequent and because laboratory results are not routinely obtained for these encounters, objective physical exam findings and laboratory results other than the GI panel were not recorded. A patient was considered to have a history of recent travel if the diarrhea started during or within 30 days after documented travel outside of Canada, Western Europe, and the United States. A history of HIV, inflammatory bowel disease (IBD), or transplant (including renal, liver, heart, lung, or hematopoietic stem cell) was considered present if it was documented in the chart at any time before or during the associated visit. Other clinical scenarios consistent with immunosuppression were active use of immunosuppressive medications, including those used for IBD, organ transplant, chemotherapy, or the equivalent of 20 mg of prednisone of glucocorticoids. Empiric antibiotic treatment was defined as any prescription of antimicrobials after the initial encounter documentation and before the availability of the results of stool testing. Subsequent notes of the ordering provider or department of that provider after the date of the test result were also reviewed to determine treatment decision-making. The study was approved by the ethical review board of the University of Virginia.

### Laboratory Testing

Stool was collected at outside clinical laboratories and transported to the central laboratory for processing daily. Stool was placed in Cary-Blair Transport Media (Remel, Lenexa, KS) at the time of collection or at the time of receipt in the central laboratory and was processed within 24 hours. For outpatients, the *Clostridioides difficile* result on the panel was suppressed.

### Interpretation of Test Results

We classified positive test results in 3 ways: (a) detection of any pathogen included on the card; (b) detection of a pathogen for which antimicrobial therapy was indicated (“pathogen warranting antimicrobial therapy”); and (c) detection of a pathogen that could lead to a change in management (“clinically-relevant pathogen”), which additionally included detection of STEC or detection of viral pathogens in immunocompromised patients, where titration of immunosuppression is often indicated (Table 1). We did not consider avoidance or discontinuation of inappropriate antibiotics to be a sufficient reason for a detection to be considered clinically relevant. Classifications for each pathogen were determined before chart review by 2 board-certified infectious diseases clinicians at our institution (J.A.P., A.J.M.). To assess the performance of the IDSA guidelines in identifying patients with clinically relevant pathogens in the outpatient setting, including in patient encounters by telephone only, we dropped signs of sepsis as a criterion and considered any report of abdominal pain to be sufficient to warrant testing, as the severity of abdominal pain could not be determined. Finally, because the indications for antimicrobial therapy can be equivocal, we performed a sensitivity analysis using a less restrictive definition of clinically relevant detection, which additionally included *Campylobacter* in the setting of >7 days of diarrhea, enterotoxigenic *E. coli* (ETEC) or enteroaggregative *E. coli* (EAEC) with no other pathogen detected in the setting of >7 days of diarrhea, and *Cryptosporidium* in a patient with >14 days of diarrhea.

**Table 1.** Pathogens Detected by the FilmArray GI Panel and Study Definitions of Pathogens Warranting Antimicrobial Therapy and Clinically Relevant Pathogens

	Pathogen Warranting Antimicrobial Therapy	Clinically Relevant Pathogen [7]
Bacteria		
*Campylobacter* spp.	Dysentery or immunocompromised	Dysentery or immunocompromised
Enteroaggregative *E. coli*	No	No
Enteropathogenic *E. coli*	No	No
Enterotoxigenic *E. coli*	No	No
*Plesiomonas shigelloides*	Immunocompromised	Immunocompromised
*Salmonella*	Age >50 y or immunocompromised	Age >50 y or immunocompromised
Shiga toxin-producing *E. coli*	No	Yes^b^
*Shigella*/enteroinvasive *E. coli*	Yes	Yes
*Vibrio* spp.	Yes	Yes
*Yersinia enterocolitica*	Yes	Yes
Viruses		
Adenovirus 40/41	No	Immunocompromised^a^
Astrovirus	No	Immunocompromised^a^
Norovirus GI/GII	No	Immunocompromised^a^
Rotavirus	No	Immunocompromised^a^
Sapovirus	No	Immunocompromised^a^
Protozoa		
*Cryptosporidium*	Immunocompromised	Immunocompromised
*Cyclospora cayetanensis*	Yes	Yes
*Entamoeba histolytica*	Yes	Yes
*Giardia*	Yes	Yes

Abbreviation: GI, gastrointestinal.

^a^Reduction in immunosuppression indicated.

^b^Withholding antibiotics indicated.

### Data Analysis

To assess for differences in characteristics of the study population between groups, we used the chi-square test for dichotomous variables and the 2-sided Student *t* test for continuous variables. Confidence intervals were derived for test characteristics using a binomial distribution. All statistical analysis was performed using R software, version 3.4.1 (Foundation for Statistical Computing).

## RESULTS

A total of 943 tests were sent from unique adult outpatients between March 23, 2015, and July 18, 2016. Of these, 314 were excluded for the following reasons: no associated visit or telephone encounter (n = 132), test was completed more than 72 hours after the associated visit or telephone encounter (n = 132), test was performed for screening before fecal transplant (n = 25), and test was sent for reason other than diarrhea (n = 25). The remaining 629 patients were included in the analysis, of whom 53 (8.4%) had a telephone encounter only. The majority of patients had a duration of diarrhea >14 days when the test was ordered (68.4%) (Table 2). There were 107 tests sent in immunocompromised patients and 22 from patients with a recent history of travel. Among patients with a recent travel history and a duration of diarrhea documented, 15/20 (75.0%) had a duration >14 days. Abdominal pain was the most common accompanying symptom but was less frequently described by immunocompromised patients (38.3% vs 54.4%; *P* = .003). A pathogen was detected in 127 of 629 specimens (20.2%), of which 18/127 (14.2%) had more than 1 pathogen detected. Enteropathogenic *E. coli* (EPEC) was the most commonly detected pathogen (47/629, 7.5%), followed by norovirus (24, 3.8%), EAEC (14, 2.2%), *Campylobacter* (9, 1.4%), and *Salmonella* (9, 1.4%) (Table 3). Norovirus was more frequently isolated from immunocompromised patients, whereas EAEC, *Campylobacter*, *Salmonella*, *Cryptosporidium*, and astrovirus were more frequently isolated from patients with acute diarrhea. Clinically relevant pathogens were more common in immunocompromised patients (19/107, 17.8%, vs 14/522, 2.7%; *P* < .001). Among travelers, the most common pathogens detected were EPEC (5/22, 22.7%), EAEC (3/22, 13.6%), and *Giardia* (2/22, 9.1%). Of the 18 patients with more than 1 pathogen detected, 14 (77.8%) had EAEC, EPEC, or both detected, 2 had both norovirus and *Giardia*, 1 had *Campylobacter* and *Plesiomonas*, and 1 had ETEC and STEC.

**Table 2.** Characteristics of the Study Population

		Overall (n = 629)	Immunocompetent (n = 522)	Immunocompromised (n = 107)	*P* Value^d^
Demographics	Age, y	53.2 ± 18.3	54.1 ± 18.7	48.8 ± 15.7	.011
	Recent antibiotic exposure	80 (12.7)	62 (11.9)	18 (16.8)	.201
	Recent travel	22 (3.5)	22 (4.2)	0 (0)	.021
	HIV	11 (1.7)	N/A	11 (10.3)	N/A
	Transplant	40 (6.4)	N/A	40 (37.4)	N/A
	Other immunosuppression	56 (8.9)	N/A	56 (52.3)	N/A
	Inflammatory bowel disease	61 (9.7)	24 (4.6)	37 (34.6)	<.001
Clinical	Duration of diarrhea, d^a^				.137
Characteristics	<3	53 (8.8)	39 (7.8)	14 (14.4)	
	3–7	75 (12.5)	67 (13.3)	8 (8.2)	
	7–14	61 (10.2)	52 (10.4)	9 (9.3)	
	>14	410 (68.4)	344 (68.5)	66 (68)	
	Vomiting	76 (12.1)	65 (12.5)	11 (10.3)	.627
	Abdominal pain	325 (51.7)	284 (54.4)	41 (38.3)	.003
	Fecal urgency	46 (7.3)	35 (6.7)	11 (10.3)	.220
	Tenesmus	4 (0.6)	3 (0.6)	1 (0.9)	.527
	Flatulence	18 (2.9)	16 (3.1)	2 (1.9)	.751
	Subjective fever	49 (7.8)	43 (8.2)	6 (5.6)	.432
	Blood in stool	78 (12.4)	76 (14.6)	2 (1.9)	<.001
Pathogen	Any pathogen	127 (20.2)	99 (19)	28 (26.2)	.112
Detection	Pathogen warranting antimicrobial therapy^b^	18 (2.9)	14 (2.7)	4 (3.7)	.526
	Clinically relevant pathogen^c^	33 (5.2)	14 (2.7)	19 (17.8)	<.001
Treatment	Empiric antimicrobials given	33 (5.2)	33 (6.3)	0 (0)	.003
	Antimicrobials given after test result	50 (7.9)	40 (7.7)	10 (9.3)	.557

Data are presented as No. (%) for dichotomous variables and mean ± SD for continuous variables.

^a^n = 599.

^b^Pathogen for which antimicrobial therapy is indicated.

^c^Pathogen for which a change in management is indicated (includes titration of immunosuppressive medications and withholding antibiotics for shiga toxin–producing *Escherichia coli*).

^d^Test for difference between immunocompromised and immunocompetent patients; Fisher exact test for dichotomous variables and 2-sided *t* test for continuous variables.

**Table 3.** Pathogen Detection by the FilmArray GI Panel

	Overall (n = 629)	Immunocompetent (n = 522)	Immunocompromised (n = 107)	*P* Value^a^	Acute (≤14 d; n = 189)	Persistent (>14 d; n = 410)	*P* Value^b^
Any pathogen	127 (20.2)	99 (19.0)	28 (26.2)	.112	61 (32.3)	61 (14.9)	<.001
Pathogen warranting antimicrobial therapy	18 (2.9)	14 (2.7)	4 (3.7)	.526	12 (6.3)	6 (1.5)	.003
Clinically relevant pathogen	33 (5.2)	14 (2.7)	19 (17.8)	<.001	16 (8.5)	16 (3.9)	.030
Enteropathogenic *E. coli*	47 (7.5)	37 (7.1)	10 (9.3)	.420	16 (8.5)	30 (7.3)	.623
Norovirus GI/GII	24 (3.8)	12 (2.3)	12 (11.2)	<.001	9 (4.8)	15 (3.7)	.509
Enteroaggregative *E. coli*	14 (2.2)	13 (2.5)	1 (0.9)	.483	8 (4.2)	5 (1.2)	.030
*Campylobacter* spp.	9 (1.4)	8 (1.5)	1 (0.9)	1.000	6 (3.2)	2 (0.5)	.014
*Salmonella*	9 (1.4)	9 (1.7)	0 (0)	.370	6 (3.2)	3 (0.7)	.031
Sapovirus	7 (1.1)	5 (1.0)	2 (1.9)	.340	4 (2.1)	2 (0.5)	.082
*Giardia*	6 (1.0)	4 (0.8)	2 (1.9)	.271	2 (1.1)	4 (1)	1.000
*Plesiomonas shigelloides*	6 (1.0)	5 (1.0)	1 (0.9)	1.000	3 (1.6)	2 (0.5)	.183
*Cryptosporidium*	6 (1.0)	6 (1.1)	0 (0)	.596	6 (3.2)	0 (0)	.001
Enterotoxigenic *E. coli*	5 (0.8)	4 (0.8)	1 (0.9)	1.000	2 (1.1)	3 (0.7)	.653
Astrovirus	5 (0.8)	4 (0.8)	1 (0.9)	1.000	5 (2.6)	0 (0)	.003
*Cyclospora cayetanensis*	2 (0.3)	2 (0.4)	0 (0)	1.000	2 (1.1)	0 (0)	.099
*Shigella*/enteroinvasive *E. coli*	1 (0.2)	1 (0.2)	0 (0)	1.000	1 (0.5)	0 (0)	.316
Adenovirus	1 (0.2)	1 (0.2)	0 (0)	1.000	0 (0)	1 (0.2)	1.000
*Vibrio* spp.	1 (0.2)	1 (0.2)	0 (0)	1.000	0 (0)	1 (0.2)	1.000
Shiga toxin–producing *Escherichia coli*	1 (0.2)	0 (0)	1 (0.9)	.170	0 (0)	1 (0.2)	1.000
*Yersinia enterocolitica*	1 (0.2)	1 (0.2)	0 (0)	1.000	1 (0.5)	0 (0)	.316

No. (%) is shown for dichotomous variables.

Abbreviation: GI, gastrointestinal.

^a^Fisher’s exact test for the difference between immunocompromised and immunocompetent patients.

^b^Fisher’s exact test for the difference between patients with acute and persistent diarrhea.

Because IDSA guidelines recommend testing for all immunocompromised patients and because the prevalence of clinically relevant pathogens was substantially higher in that population, we then looked at the subgroup of immunocompetent patients (n = 522) to identify clinical characteristics associated with clinically relevant pathogens (Table 4). Patients with a pathogen warranting antimicrobial therapy detected had a shorter duration of diarrhea (*P* = .002) and were more likely to report a fever (*P* = .022). Abdominal pain was also more common in the setting of a pathogen warranting antimicrobial therapy; however, this difference was not statistically significant. There was no relationship between the presence of blood in stool or empiric antimicrobial therapy and the presence of a pathogen warranting antimicrobial therapy. The presence of at least 1 of the IDSA guideline criteria for diagnostic testing, namely fever, abdominal pain, or blood in stool, was associated with detection of a clinically relevant pathogen (13/14, 92.9%, vs 306/508, 60.2%; *P* = .012). No patients with diarrhea without either an enteric symptom (abdominal pain, nausea, vomiting, fecal urgency, tenesmus, or flatulence) or subjective fever had a clinically relevant pathogen detected (0/174, 0%, vs 14/348, 4.0%, with either an enteric symptom or subjective fever; *P* = .007).

**Table 4.** Characteristics of Immunocompetent Patients With and Without Pathogens Warranting Antimicrobial Therapy Detected

		No Pathogen Warranting Antimicrobial Therapy Detected (n = 508)	Pathogen Warranting Antimicrobial Therapy Detected (n = 14)	*P* Value^b^
Demographics	Age, y	54.1 ± 18.8	52.6 ± 15.1	.700
	Recent antibiotic exposure	60 (11.8)	2 (14.3)	.677
	Recent travel	19 (3.7)	3 (21.4)	.018
	Inflammatory bowel disease	24 (4.7)	0 (0)	1.000
Clinical	Duration of diarrhea, d^a^			.002
Characteristics	<3	38 (7.8)	1 (7.1)	
	3–7	63 (12.9)	4 (28.6)	
	7–14	47 (9.6)	5 (35.7)	
	>14	340 (69.7)	4 (28.6)	
	Vomiting	64 (12.6)	1 (7.1)	1.000
	Abdominal pain	273 (53.7)	11 (78.6)	.100
	Fecal urgency	35 (6.9)	0 (0)	.614
	Tenesmus	3 (0.6)	0 (0)	1.000
	Flatulence	16 (3.1)	0 (0)	1.000
	Subjective fever	39 (7.7)	4 (28.6)	.022
	Blood in stool	74 (14.6)	2 (14.3)	1.000
	Abdominal pain, subjective fever, or blood in stool	306 (60.2)	13 (92.9)	.012
Treatment	Empiric antimicrobials given	32 (6.3)	1 (7.1)	.604
	Antimicrobials given after test result	30 (5.9)	10 (71.4)	<.001

Data are presented as No. (%) for dichotomous variables and mean ± SD for continuous variables.

^a^n = 502.

^b^Fisher’s exact test for dichotomous variables and 2-sided *t* test for continuous variables.

We then assessed the test characteristics of the IDSA guideline–based criteria for diagnostic testing in the entire study population, including immunocompromised patients. These criteria had a sensitivity of 97.0% (95% confidence interval [CI], 84.2%–99.9%), a specificity of 33.9% (95% CI, 30.1%–37.8%), a negative predictive value of 99.5% (95% CI, 97.3%–100.0%), and a positive predictive value of 7.5% (95% CI, 5.2%–10.4%) (Table 5). Adherence with this recommendation would have avoided testing in 203/522 immunocompetent patients (38.9%) and 203/629 overall (32.3%) while still recommending testing for 32/33 patients (97.0%) with a clinically relevant pathogen. Among immunocompetent patients, antimicrobial therapy was prescribed in response to the test result in 40/522 (7.7%) and was more frequently prescribed in patients with detection of a pathogen warranting antimicrobial therapy (10/14, 71.4%, vs 30/508, 5.9%; *P* < .001). Application of the IDSA criteria would have avoided testing in 7/30 patients (23.3%) for whom antibiotics were prescribed despite detection of a pathogen that did not warrant therapy. The majority of these prescriptions (20/30, 66.7%) were for treatment of either EAEC or EPEC.

**Table 5.** Testing Recommendation Based on IDSA Guidelines^a^ and Detection of Clinically Relevant Pathogens in the Study Population

	No Clinically Relevant Pathogen Detected, No. (%)	Clinically Relevant Pathogen Detected, No. (%)	Total No. (%)
Test^a^	394 (66.1)	32 (97.0)	426 (67.7)
Don’t test	202 (33.9)	1 (3.0)	203 (32.3)
Total	596	33	629

Abbreviation: IDSA, Infectious Diseases Society of America.

^a^Recommendation to test if the patient is (a) immunocompromised or (b) describes subjective fever, abdominal pain, or blood in stool.

Recognizing the lack of clinical consensus about the role of antibiotic treatment for several enteropathogens, we performed a sensitivity analysis using a less restrictive definition of a clinically relevant detection. Specifically, this definition broadened the role of antibiotic treatment to include patients with *Campylobacter* and >7 days of symptoms, ETEC or EAEC with both >7 days of symptoms and no other pathogen identified, and C*ryptosporidium* with >14 days of symptoms. Using this definition, the sensitivity of IDSA guideline–based criteria was 88.4% (95% CI, 74.9%–96.1%), the specificity was 33.8% (95% CI, 30.0%–37.8%), and the negative predictive value was 97.5% (95% CI, 94.3%–99.2%).

## Discussion

In this study of adult outpatients with diarrhea at a single institution utilizing a culture-independent, multiplex molecular diagnostic platform for the detection of gastrointestinal pathogens, we validated the IDSA guidelines’ recommendations as sensitive but not specific clinical criteria for the use of diagnostic testing and showed that use of these guidelines could reduce testing by approximately one-third without reducing the clinical yield. Although these criteria do not apply to unique circumstances, such as in the context of a possible outbreak or in the management of food or health care workers, they may help reduce unnecessary use of these expensive tests in the outpatient setting.

In immunocompetent patients, the IDSA recommendation to pursue diagnostic testing in adult patients with diarrhea accompanied by at least 1 of fever, blood in stool, or abdominal pain was designed to target testing toward patients with a higher pretest probability of detection of invasive bacterial enteropathogens, namely *Salmonella*, *Shigella*, *Campylobacter*, *Yersinia*, and STEC. In our population, the guidelines’ criteria were sensitive but not specific for detection of these pathogens, suggesting that testing can safely be deferred in patients who have none of these clinical characteristics. A single patient who did not meet the IDSA criteria had a pathogen that warranted antimicrobial therapy based on our pre hoc classification, a 70-year-old male with *Salmonella*. The primary benefit of treatment of *Salmonella* diarrhea in immunocompetent adults is reduction of the risk of invasive disease in high-risk patients, including those >50 years of age, although some physicians would further restrict testing to those with additional risk factors such as atherosclerotic disease [13].

In our study, additional criteria that could potentially be used to stratify the role of diagnostic testing, such as a recent travel history and diarrhea duration, did not differentiate patients with a clinically relevant pathogen. Strikingly, the majority of testing was performed on patients with diarrhea for 14 days or longer, in whom the clinical yield was even lower. This is consistent with the assumption that many patients do not seek care for what is usually a self-limited syndrome, and thus testing was enriched in those patients with persistent symptoms, where the pretest probability of an infectious etiology is lower. This study thus does not support the use of duration-based criteria for diagnostic testing. Although empiric therapy of acute traveler’s diarrhea is supported by data demonstrating a reduction in the duration of symptoms [14, 15], the use of recent travel as a criterion for empiric therapy or even for the use of diagnostic tests was not supported in the setting of an adult outpatient clinic in the United States where the majority of patients presented with persistent diarrhea even when a travel history was documented. In this context, targeted testing for giardiasis and evaluation for postinfectious irritable bowel syndrome would likely be a more rational approach [16, 17].

These data did support the broad application of diagnostic testing to immunocompromised patients with diarrhea, as these patients showed fewer signs and symptoms of infection, including abdominal pain and blood in stool. Furthermore, immunocompromised patients had a significantly higher yield of pathogens that warranted a change in clinical management. We considered detection of viral agents of gastroenteritis as possibly changing clinical management due to the potential benefit of titrating the degree of immunosuppression. This is particularly relevant for norovirus, sapovirus, and astrovirus, which have been associated with chronic diarrhea in immunocompromised patients [18–20]. Treatment of chronic norovirus and sapovirus gastroenteritis with nitazoxanide may also be beneficial, though we did not classify these as pathogens warranting antimicrobial therapy in this study [21].

Application of these criteria for diagnostic testing would only have averted a minority of the antimicrobial therapy that we did not consider clinically warranted. The restriction of testing for pathogens that never require treatment, as well as clear guidance for physicians, may be more effective for antimicrobial stewardship than strategies that reduce testing. Several additional patients with detections that some physicians would consider treating did not meet the IDSA criteria, as detailed in the sensitivity analysis. However, our clinical experience has generally not supported a role for antibiotic therapy for patients with *Campylobacter* and diarrheagenic *E. coli* based on the duration of symptoms alone. The testing and reporting of EPEC present a particular challenge for clinicians, as it is unclear whether EPEC is causative when detected in patients with diarrhea [22]. In our chart review, we anecdotally noted frequent use of the infectious diseases consult service to determine the need for treatment of these pathogens. We did not consider *Clostridioides difficile* infection in this study. However, because of the high pretest probability of this organism in specific populations, for example, those with recent antibiotic exposure, we would not exclude diagnostic testing based on the IDSA criteria alone. Further, the value of single-step PCR testing for *C. difficile* has been called into question [23].

The rational and efficient use of diagnostic testing is an important consideration in efforts to improve value in health care, and evidenced-based guidance for the reduction of inappropriate testing is of significant value. Multiplex diagnostic panels for diarrhea are expensive, whereas the syndrome, especially in immunocompetent patients, is frequently self-limited and requires no treatment. Indeed, Medicare has recently restricted reimbursement for enteric pathogen panels in immunocompetent patients in 7 states in the southeastern United States, with coverage determinations for the rest of the country pending [12].

This study had several limitations. As with any chart review, it is possible that the information discussed with the patient may not have made it into the chart in its entirety. However, our estimation of the sensitivity of the IDSA guidelines should thus be conservative. Additionally, we did not include objective physical exam findings (such as abdominal tenderness) because telephone consultations were common for this complaint in the outpatient setting. The incorporation of objective findings as well as limiting the testing criteria to the subset of severe abdominal pain may have increased the specificity, with an uncertain trade-off of reduced sensitivity. A prospectively enrolled study would be needed to further evaluate this. Because our study question required clear, pre hoc identification of how pathogens would change clinical management, we determined these outcomes before data collection and analysis based on consensus between 2 infectious diseases physicians at our institution. In reality, treatment decisions are often less clear cut. As an example, some recommend limiting antimicrobial therapy for shigellosis to immunocompromised patients and those with more severe disease [24]. However, we believe that our treatment recommendations are generally consistent with clinical practice, and the negative predictive value of the IDSA criteria remained high in a sensitivity analysis that incorporated additional detections that some providers would consider clinically relevant.

In summary, a rapid and highly sensitive molecular diagnostic panel had a relatively low clinical yield in adult outpatients with diarrhea in our institution, and application of the testing criteria elucidated in the 2017 IDSA guidelines could reduce testing without reducing the clinical yield. The uniform use of such criteria should be considered for increasing the value of these tests in such settings.
